# The Long-Term, Real-World Effects of Oxybutynin on Pressure Reservoir Function in the Neurogenic Bladder after Spinal Cord Injury: A Retrospective Cohort Study

**DOI:** 10.3390/jcm13154514

**Published:** 2024-08-02

**Authors:** Sirintip Boonjaraspinyo, Jittima Saengsuwan, Patpiya Sirasaporn, Bandit Thinkhamrop

**Affiliations:** 1 Department of Community, Family and Occupational Medicine, Faculty of Medicine, Khon Kaen University, Khon Kaen 40002, Thailand; sboon@kku.ac.th; 2 Epidemiology and Biostatistics Program, Faculty of Public Health, Khon Kaen University, Khon Kaen 40002, Thailand; 3 Department of Rehabilitation Medicine, Faculty of Medicine, Khon Kaen University, Khon Kaen 40002, Thailand; spatpiya@kku.ac.th; 4 Department of Epidemiology and Biostatistics, Faculty of Public Health, Khon Kaen University, Khon Kaen 40002, Thailand; bandit@kku.ac.th

**Keywords:** neurogenic bladder, oxybutynin, spinal cord injury

## Abstract

**Background/Objectives**: Data on the long-term effects of oxybutynin in patients with neurogenic bladder (NGB) due to spinal cord injury (SCI) are limited. This study aimed to evaluate the long-term effects of oxybutynin and the combination of oxybutynin with trospium in these patients, under real-world medical conditions. **Methods**: A total of 107 patients with NGB due to SCI were included. The mean treatment duration was 2.8 years ± 0.8 years. The patients were categorized into three groups: (1) low-dose oxybutynin (5–15 mg/day), (2) high-dose oxybutynin (20–40 mg/day), and (3) oxybutynin combined with trospium. The main outcomes were maximal detrusor pressure (MDP) and cystometric bladder capacity (CBC). Both were assessed at baseline and at three subsequent follow-up visits. Generalized estimation equation models were used to estimate the overall mean reduction in MDP and CBC for each group. **Results**: The overall adjusted mean reduction from baseline of MDP in groups 1, 2, and 3 were 2.5 (95% CI: −5.4 to 10.4; *p* = 0.540), 16.9 (95% CI: 4.4 to 29.4; *p* = 0.008), and 21.9 (95% CI: 4.1 to 39.8; *p* = 0.016) cmH_2_O, respectively. For the CBC, the mean reduction was not significant in any group at any visit, nor were the overall mean reductions. **Conclusions**: These findings suggest that high-dose oxybutynin and oxybutynin–trospium combination achieve a significant long-term reduction in MDP in patients with NGB after SCI. The effects were sustained across all three follow-up periods.

## 1. Introduction

The global prevalence of spinal cord injury (SCI) ranges from 15 to 40 per million [1,2,3]. In Thailand, reported incidence rates for SCI vary between 5.8 and 23.0 per million [4,5]. Most individuals diagnosed with SCI (70–84%) experience bladder dysfunction [6,7], and bladder problems are among the top health priorities for these individuals [8]. 

SCIs commonly compromise bladder function, often resulting in a range of symptoms associated with overactive bladder, such as frequency or urgency. Most patients (72.2–95%) with suprasacral lesions have detrusor overactivity (DO) and/or detrusor sphincter dyssynergia (DSD) [9,10,11], leading to high detrusor pressure and urinary retention. Conversely, patients with sacral lesions may experience an acontractile bladder. However, studies have shown that a significant proportion (28–100%) of patients with sacral injuries have low bladder compliance and a substantial subset (4.3–90%) have high detrusor leak point pressure [9,11,12]. Nonetheless, the mechanisms underlying decreased compliance remain uncertain [13]. All of the above-mentioned abnormalities contribute to various urological complications, including upper urinary tract deterioration and renal dysfunction [14].

One of the critical goals in managing neurogenic detrusor overactivity (NDO) is to facilitate low-pressure storage and efficient bladder emptying at low detrusor pressure to prevent upper and lower urinary tract complications from high detrusor pressures [6,15]. Multiple effective treatment options are available, with the first line of therapy often involving anticholinergic medications [16]. These drugs, specifically the muscarinic receptor antagonists, are the main pharmacological treatment for overactive bladder syndrome (OAB) [17,18]. Currently, several anticholinergics are approved for OAB management [19]. This study focuses on oxybutynin and trospium, two drugs widely used in Thailand. Multiple studies have demonstrated the effectiveness of oral oxybutynin and trospium in controlling OAB [20,21]. 

While studies have shown the benefits of anticholinergic drugs for neurogenic bladder (NGB) in a 1-year period [22,23], there is a critical lack of clear evidence on how these medications perform in real-world, longer-term situations. Considering that individuals with NDO exhibit morphological changes in the bladder wall, such as inflammatory infiltration, edema, and fibrosis over time [24], there is a possibility that the efficacy of anticholinergic medication could diminish with prolonged use. This study addresses this knowledge gap by evaluating the long-term effects of oxybutynin and oxybutynin combined with trospium on maximal detrusor pressure (MDP) reduction in patients with NGB after SCI. This study aimed to assess the drugs’ effectiveness with a focus on long-term treatment effects, providing valuable data for long-term NGB management.

## 2. Materials and Methods

### 2.1. Design and Setting

This retrospective cohort study was conducted using hospital-based data from a tertiary care hospital in northeastern Thailand between 1999 and 2016. A pre-defined database was available for this study [25]. Ethical approval for use of the data was obtained from the Center for Ethics in Human Research, Khon Kaen University (reference number HE651472). 

### 2.2. Patient Population

The medical records of patients were chronologically selected based on the following inclusion criteria: (i) NGB confirmed by history and urodynamic assessment due to SCI, (ii) age 18–80 years, (iii) treatment with oral oxybutynin IR or oral trospium IR for neurogenic bladder, (iv) first treatment within 5 years of SCI, and (v) treatment duration of at least 6 months, between every follow-up. The exclusion criterion was patients concurrently receiving multiple neurogenic bladder medications, other than oxybutynin and trospium. Patients who met the inclusion criteria were initially included in the study cohort. Subsequently, patients with medication changes, those lost to follow-up, or those lacking MDP data were excluded from the analysis. The neurological level of injury and the degree of impairment after SCI were documented based on the International Standards for Neurological Classification of Spinal Cord Injury (ISNCSCI) [26]. 

### 2.3. Urodynamic Study

Urodynamics were conducted with patients in the supine position, following a standardized procedure as described in a previous study [25]. Maximum detrusor pressure (MDP) was defined as the highest detrusor pressure measured during filling cystometry, while cystometric bladder capacity (CBC) was determined as the bladder volume at the end of the filling cystometrogram [27]. 

### 2.4. Study Outcomes

The primary outcome of the study was the reduction in MDP following antimuscarinic treatment. An MDP decrease to ≤40 cmH_2_O was considered clinically successful [28]. The secondary outcome was the increase in CBC after antimuscarinic treatment. The long-term treatment effect was evaluated by assessing outcomes over three repeated follow-up periods for MDP or CBC. These outcomes were measured at baseline (before the first treatment) and at each follow-up (FU) visit at FU1, FU2, and FU3 (with a minimum treatment duration of 6 months). 

### 2.5. Statistical Analysis

The analysis was conducted using STATA software (version 18.0 for Windows; StataCorp LLC, College Station, TX, USA) to perform a generalized estimating equation (GEE) analysis. This analysis assessed the reduction in MDP or CBC for each treatment group separately, as the treatment was preselected at baseline based on the clinical data of patients, which influenced the outcome. The patients were categorized into three treatment groups: (1) low-dose oxybutynin (5–15 mg/day), (2) high-dose oxybutynin (20–40 mg/day), and (3) oxybutynin combined with trospium. The outcomes are presented as means ± standard deviations (SD) at baseline and follow-up visits 1, 2, and 3. Mean differences, 95% confidence intervals (CI), and *p*-values were calculated using GEE, with adjustments made for age (in years), sex (women/men), level of SCI (suprasacral/sacral), completeness of lesion (yes/no), and indwelling urinary catheter use (yes/no). Suprasacral lesion was defined as an injury to the spinal cord above the level of the sacral cord and roots [13]. Complete injury was defined as no preservation of sensory or motor function in the sacral segment S4–S5 [26]. Statistical significance was set at a two-tailed *p*-value < 0.05.

## 3. Results

**Figure 1:**
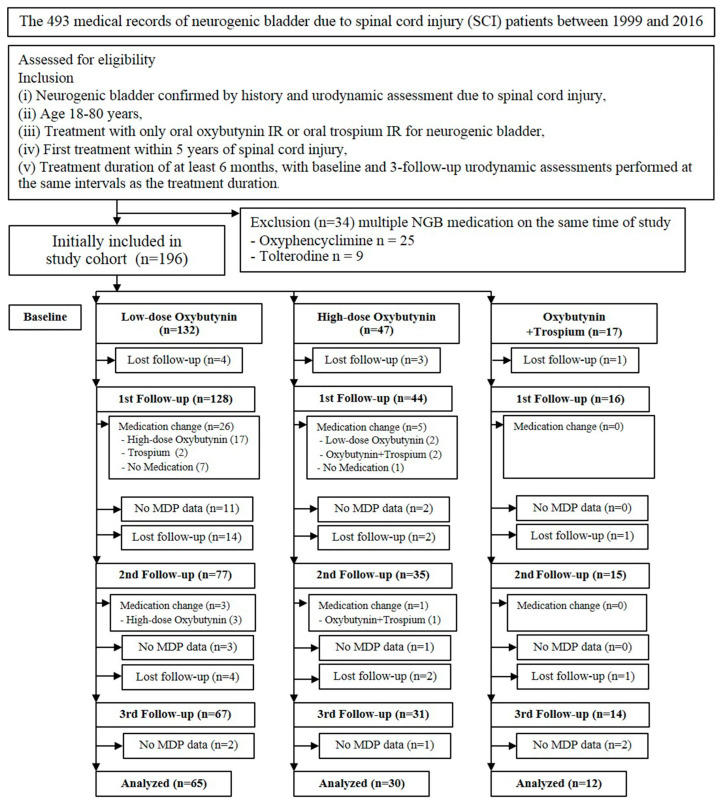
Flow diagram of the study cohort.

One hundred and ninety-six participants were initially included in the study cohort. There were 132 patients in the low-dose oxybutynin group, 47 patients in the high-dose oxybutynin group, and 17 patients in the combined oxybutynin and trospium group. In the low-dose oxybutynin group, approximately half of the patients were excluded due to being lost to follow-up (16.7%), medical changes (22%), and missing MDP data (12.1%). In the high-dose oxybutynin group, one-third of the patients were excluded due to being lost to follow-up (14.9%), medical changes (12.8%), and missing MDP data (8.5%). In the combined oxybutynin and trospium group, nearly one-third were excluded due to being lost to follow-up (17.8%) and missing MDP data (11.8%); none of the patients in this group had to change medication. We finally analyzed 107 eligible participants who completed all the sessions and did not have to change medication. In total, 65 patients were in the low-dose oxybutynin group (5–15 mg/day), 30 patients were in the high-dose oxybutynin group (20–40 mg/day mg), and 12 patients were in the oxybutynin combined with trospium group (oxybutynin 10–40 mg/day and trospium 20–80 mg/day). The complete study flowchart is depicted in Figure 1.

In our clinic, physicians determined the dosage primarily based on baseline MDP; however, other factors such as lesion level, completeness of lesion, bladder management methods, and predicted risk of upper tract deterioration may also have influenced the decision. As shown in Table 1, in general, the higher the MDP, the more likely that the patients would receive high medical dosage or combined medication. Additionally, patients with a lower age at the diagnosis of SCI tended to have a higher anticholinergic dosage or combined medication, as did a higher percentage of patients with suprasacral and complete SCI. 

**Figure 2:**
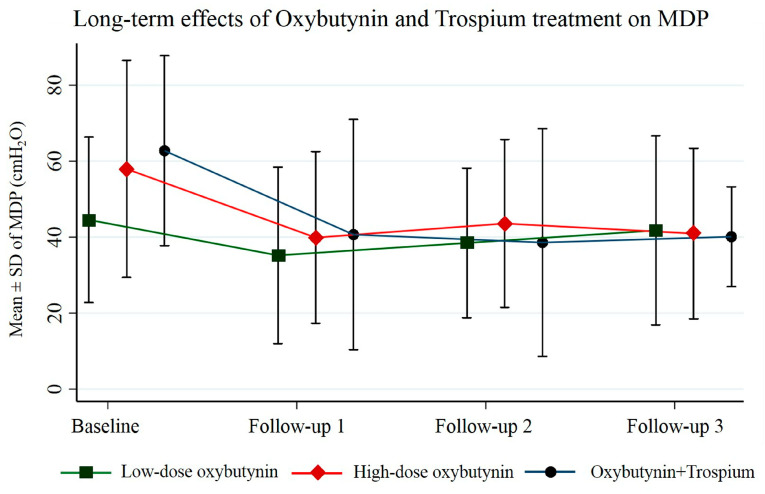
Overall long-term dose–response effects of the low-dose oxybutynin, high-dose oxybutynin, and oxybutynin combined with trospium on maximal detrusor pressure.

**Figure 3:**
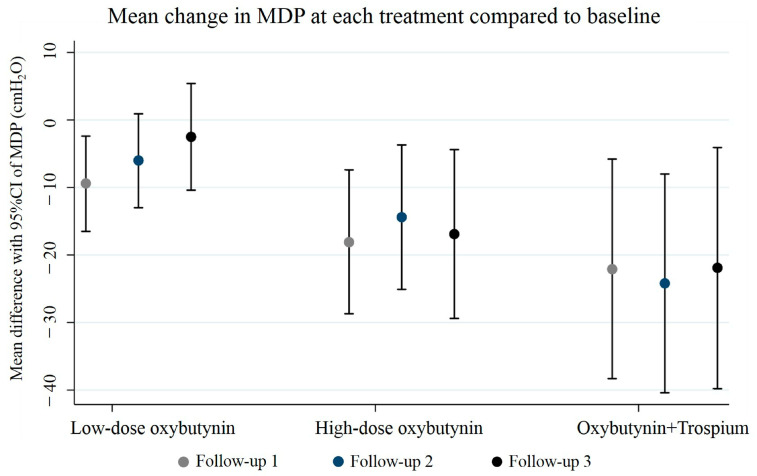
Mean difference with 95%CI of MDP at each follow-up compared to baseline, separated by treatment medication.

Overall, patients were followed up consecutively, with a mean treatment duration of 2.8 ± 0.8 years. Each follow-up interval averaged between 0.9 and 1.2 years. Our primary analysis revealed that low-dose oxybutynin treatment significantly decreased MDP by 9.4 cmH_2_O (95% CI 2.4, 16.5; *p* = 0.009) only at the first follow-up, but not at the second and third follow-ups (*p* = 0.089 and *p* = 0.540). High-dose oxybutynin treatment produced dramatic reductions in MDP in all three consecutive follow-ups: the baseline MDP was 57.9 ± 28.5 cmH_2_O, which then significantly decreased to 39.9 ± 22.6, 43.6 ± 22.1, and 40.9 ± 22.4 cmH_2_O at follow-ups 1, 2, and 3, respectively. This demonstrates a substantial and sustained reduction in MDP (adjusted mean reduction of 16.9 cmH_2_O, 95% CI 4.4, 29.4; *p* = 0.008) with high-dose oxybutynin therapy. Combination therapy with oxybutynin and trospium exhibited a trend towards a significant decrease in MDP over time in all three follow-ups. The baseline MDP was 62.8 ± 25.0 cmH_2_O, and this was progressively reduced to 40.7 ± 30.3, 38.6 ± 30.0, and 40.1 ± 13.1 cmH_2_O at follow-ups 1, 2, and 3, respectively. Long-term treatment with this combination therapy achieved a notable reduction in MDP (adjusted mean reduction of 21.9 cmH_2_O, 95% CI: 4.1, 39.8; *p* = 0.016). A significant overall reduction in MDP over time was observed among participants in the high-dose oxybutynin group (adjusted mean difference −5.2 cmH_2_O, 95% CI: −9.1, −1.2; *p* = 0.011) and the oxybutynin combined with trospium group (adjusted mean difference −7.4 cmH_2_O, 95% CI: −13.3, −1.5; *p* = 0.014). The average MDP and the overall mean MDP reduction for each group are presented in Table 2, Figure 2 and Figure 3. CBC remained remarkably stable across all treatment groups, with no significant changes observed between baseline and follow-ups 1, 2, and 3 (Table 3). The success rates for MDP control are presented in Table 4. Notably, the low-dose oxybutynin group at the last follow-up exhibited the lowest success rates for MDP (54.8%) compared to all other groups.

## 4. Discussion

This retrospective cohort study investigated the long-term effects of oxybutynin and oxybutynin combined with trospium on maximal detrusor pressure (MDP) and cystometric bladder capacity (CBC) in patients with NGB after SCI. We found that oxybutynin at the dosage of 15 mg or lower did not show a significant decrease in MDP (baseline 44.5 vs. third follow-up [FU] 41.8 cmH_2_O) and could not prevent a decrease in CBC (baseline 253.4 vs. third FU 223.7 mL). Our study demonstrated a lower MDP response to anticholinergic medication compared to multiple previous studies. For example, Madersbacher et al. showed that patients with SCI with NDO and treated with 15 mg of oxybutynin over 2–3 weeks showed a decrease in MDP of 38 cmH_2_O (82 vs. 44 cmH_2_O) and an increase in CBC of 163 mL (188 vs. 351 mL) [29]. We propose that our patients might have experienced a delay in the initiation of anticholinergic medication, leading to morphological changes in the bladder wall and a diminished response to medication. This is consistent with the study of Comperat et al., which demonstrated that bladder wall histology showed a higher degree of morphological changes such as inflammatory cell infiltration or fibrosis in patients with NDO not receiving medication [24]. In addition, it was shown that the time since SCI and the initiation of medication is a significant factor affecting the effectiveness of anticholinergic therapy [30]. The reason for the delay was due to referral time from other centers than ours, as well as a time gap for admission to the inpatient rehabilitation unit for a complete urological checkup.

We observed a reduction in MDP of 17 cmH_2_O and the maintenance of CBC in a group receiving high-dose oxybutynin over three follow-up visits, with an average duration of 2.8 years. Additionally, our study demonstrated that groups receiving high-dose oxybutynin and combined medication maintained their CBC over time. Although the CBC was maintained, there was no significant increase observed. The lower response in CBC increase may be attributed to the average delay in the initiation of anticholinergic therapy, as stated earlier. Furthermore, groups receiving high-dose oxybutynin and combined medication showed an increased percentage of patients with their MDP controlled to less than 40 cmH_2_O: from 36.7 to 66.7% in the group receiving high-dose oxybutynin and from 16.7 to 66.7% in the group receiving oxybutynin and trospium.

The low-dose oxybutynin group exhibited a potential waning effect over time. While an initial reduction in MDP was observed, a slight increase in MDP during the later follow-up assessments was noted. Bladder function is known to deteriorate over time in individuals with NGB [31]. For patients receiving low-dose therapy, their initial bladder function might not be significantly compromised. However, over time, low-dose oxybutynin might not be able to maintain an adequate reduction in MDP. 

Contrastingly, the groups receiving high-dose oxybutynin and the combination of oxybutynin and trospium achieved a significant reduction in MDP. The effectiveness of these high-dose and combination therapies was sustained across all three follow-up periods. It should be noted that physicians typically determined medication use or increased anticholinergic dosage primarily based on MDP, along with other factors such as lesion level, the completeness of the lesion, bladder management methods, and the predicted risk of upper tract deterioration. The higher the MDP, the more likely patients were to receive high medication dosages or combined medications. The significant decrease in MDP in the high-dose oxybutynin and combined oxybutynin and trospium groups is noteworthy, especially since their initial MDP conditions were more severe than those in the low-dose oxybutynin group. This finding in the long-term follow-up aligns with previous research on tolterodine, suggesting increased effectiveness with increased anticholinergic dosage in a short-term period [32].

This study has limitations inherent to its retrospective design, and the data should be interpreted with caution. Firstly, medication adherence data for each participant were unavailable. This information is crucial for accurately assessing treatment efficacy, as it can significantly influence outcomes. This study experienced an attrition rate of approximately 14.9–17.6% due to loss to follow-up, which may introduce selection bias and lead to an underestimation of the true effect of the medication. Additionally, attrition due to changes in medication may reflect treatment failure. Following treatment and subsequent follow-up, physicians may adjust dosages to optimize MDP for patients with high MDP. Treatment non-adherence or the need for medication adjustments may be the most challenging indicator of insufficient treatment. However, these medical changes could confound the relationship between the medication and the outcome. To address this, we excluded patients with medical changes to ensure that outcomes could be specifically attributed to the medication. In addition, the absence of recorded data on medication adverse effects may prevent a comprehensive assessment of potential side effects experienced by participants. Secondly, the clinical protocol for treatment selection relied solely on baseline MDP, resulting in a gradual increase in anticholinergic dosage or combined therapy in groups with higher baseline MDP. This limitation likely contributed to the need for analyzing separate treatment groups. Despite addressing these issues through separate group analysis, the relatively small sample size, particularly within the combined-therapy group, is a significant limitation. While studies focusing on this specific drug combination are scarce, a larger sample size would strengthen our understanding of treatment dynamics in this patient population. Although our study demonstrated that high-dose oxybutynin or combination therapy showed MDP reduction over nearly 3 years and a stable CBC over time, it should be noted that our study showed a lower response compared to previous short-term studies [29]. Our population showed a longer time from SCI to therapy initiation, which was due to the transfer process from the other medical centers and the appointment time for urological surveillance. Additionally, our data collection faced the limitation of ending in 2016, due to mechanical problems with the urodynamic machine.

Future research efforts should address these limitations by employing a prospective design that incorporates medication adherence monitoring and accounts for potential confounding factors influencing MDP reduction. Examining the effects of both high and low doses across all treatment groups, and comparing long-term treatment outcomes, could provide valuable insights for optimizing long-term bladder management strategies for patients with NGB after SCI.

## 5. Conclusions

These findings suggest that high-dose oxybutynin and oxybutynin–trospium combination achieve a significant long-term reduction in MDP in patients with NGB after SCI. The effects were sustained across all three follow-up periods.

## Figures and Tables

**Table 1:** The demographic characteristics and urodynamic values at baseline in the low-dose oxybutynin, high-dose oxybutynin, and oxybutynin combined with trospium groups.

Mean or % Variable	Low-Dose	High-Dose	Oxybutynin + Trospium
Number of patients	65	30	12
Age at diagnosis, years	44.9 ± 14.9	41.3 ± 13.8	33.0 ± 13.8
Sex: women/men (%)	35.4/64.6	36.7/63.3	41.7/58.3
Duration after SCI, years	1.2 ± 1.1	1.7 ± 1.5	1.2 ± 1.1
Duration of treatment, years	2.8 ± 0.8	2.8 ± 0.9	2.9 ± 0.9
Suprasacral injury (%)	89.2	90	100
Complete injury (%)	29.2	50.0	41.7
Indwelling urinary catheter (%)	41.5	33.3	25
Baseline values			
MDP (cmH_2_O)	44.5 ± 21.8	57.9 ± 28.5	62.8 ± 25.0
CBC (mL)	253.4 ± 125.6	247.8 ± 116.9	278.2 ± 176.9

Data are presented as means ± standard deviations unless otherwise specified. Abbreviations: MDP maximal detrusor pressure; CBC cystometric bladder capacity.

**Table 2:** Mean difference in maximal detrusor pressure (MDP) from Generalized Estimating Equations (GEE) Models, analyzed separately for each treatment group.

MDP (cmH_2_O)	Low-Dose Oxybutynin	High-Dose Oxybutynin	Oxybutynin + Trospium
n	65	30	12
**Baseline (BL)**	**44.5 ± 21.8**	**57.9 ± 28.5**	**62.8 ± 25.0**
**Follow-up 1 (FU1)**	**35.2 ± 23.2**	**39.9 ± 22.6**	**40.7 ± 30.3**
Time from BL toFU1, year	1.0 ± 0.4	0.9 ± 0.3	1.1 ± 0.5
Mean difference (95% CI)	−9.5 (−16.6, −2.5)	−18.1 (−28.8, −7.3)	−22.1 (−38.2, −5.9)
*p*-value	0.008 *	0.001 *	0.007 *
Adjusted mean difference (95% CI)	−9.4 (−16.5, −2.4)	−18.1 (−28.7, −7.4)	−22.1 (−38.3, −5.8)
*p*-value	0.009 *	0.001 *	0.008 *
**Follow-up 2 (FU2)**	**38.5 ± 19.7**	**43.6 ± 22.1**	**38.6 ± 30.0**
Time from FU1 to FU 2, year	1.1 ± 0.4	1.2 ± 0.5	1.0 ± 0.5
Mean difference (95% CI)	−6.2 (−13.1, 0.8)	−14.5 (−25.3, −3.6)	−24.2 (−40.3, −8.0)
*p*-value	0.081	0.009 *	0.003 *
Adjusted mean difference (95% CI)	−6.0 (−13.0,0.9)	−14.4 (−25.1,−3.7)	−24.2 (−40.4,−8.0)
*p*-value	0.089	0.009 *	0.003 *
**Follow-up 3 (FU3)**	**41.8 ± 24.9**	**40.9 ± 22.4**	**40.1 ± 13.1**
Time from FU2 to FU3, year	1.0 ± 0.4	1.1 ± 0.4	1.0 ± 0.5
Mean difference (95% CI)	−2.0 (−9.8, 5.9)	−17.9 (−30.5, −5.2)	−21.9 (−39.6, −4.2)
*p*-value	0.628	0.006 *	0.015 *
Adjusted mean difference (95% CI)	−2.5 (−10.4, 5.4)	−16.9 (−29.4, −4.4)	−21.9 (−39.8, −4.1)
*p*-value	0.540	0.008 *	0.016 *
**Overall changes**			
Mean difference (95% CI)	−0.8 (−3.2, 1.7)	−5.4 (−9.4, −1.4)	−7.4 (−13.2, −1.5)
*p*-value	0.553	0.008 *	0.014 *
Adjusted mean difference (95% CI)	−0.9 (−3.4, 1.6)	−5.2 (−9.1, −1.2)	−7.4 (−13.3, −1.5)
*p*-value	0.492	0.011 *	0.014 *

* *p*-value < 0.05.

**Table 3:** Mean difference in cystometric bladder capacity (CBC) from Generalized Estimating Equations (GEE) Models, analyzed separately for each treatment group.

CBC (mL)	Low-Dose Oxybutynin	High-Dose Oxybutynin	Oxybutynin + Trospium
n	65	30	12
**Baseline**	**253** **.** **4 ± 125** **.** **6**	**247** **.** **8 ± 116** **.** **9**	**278** **.** **2 ± 176** **.** **9**
**Follow-up 1**	**265** **.** **5 ± 134** **.** **0**	**234** **.** **6 ± 122** **.** **6**	**267** **.** **1 ± 166** **.** **8**
Mean difference (95% CI)	10.5 (−27.6, 48.7)	−13.2 (−59.7, 33.3)	−11.1 (−99.4, 77.2)
*p*-value	0.589	0.578	0.806
Adjusted mean difference (95% CI)	10.9 (−27.3, 49.2)	−13.2 (−59.5, 33.1)	−11.1 (−98.1, 75.9)
*p*-value	0.575	0.576	0.803
**Follow-up 2**	**260** **.** **3 ± 135** **.** **5**	**213** **.** **5 ± 92** **.** **0**	**248** **.** **0 ± 97** **.** **3**
Mean difference (95% CI)	6.1 (−31.3, 43.5)	−34.3 (−81.8, 13.2)	−30.2 (−118.5, 58.2)
*p*-value	0.749	0.157	0.503
Adjusted mean difference (95 % CI)	5.6 (−31.9, 43.1)	−34.0 (−81.4, 13.3)	−30.2 (−117.2, 56.8)
*p*-value	0.769	0.158	0.497
**Follow-up 3**	**223** **.** **7 ± 142** **.** **7**	**242** **.** **9 ± 140** **.** **6**	**270** **.** **9 ± 186** **.** **2**
Mean difference (95% CI)	−29.8 (−72.7, 13.1)	−8.0 (−62.0, 46.1)	−4.2 (−101.2, 92.9)
*p*-value	0.174	0.772	0.933
Adjusted mean difference (95% CI)	−29.1 (−72.1, 14.0)	−6.9 (−60.8, 47.0)	−7.8 (−103.8, 88.1)
*p*-value	0.186	0.801	0.873
**Overall changes**			
Mean difference (95% CI)	−7.3 (−20.5, 6.0)	−6.5 (−23.2, 10.2)	−4.5 (−34.8, 25.9)
*p*-value	0.283	0.445	0.772
Adjusted mean difference (95% CI)	−7.2 (−20.5, 6.1)	−6.2 (−22.9, 10.4)	−5.5 (−35.5, 24.4)
*p*-value	0.29	0.465	0.718

**Table 4:** Success rate (%) of urodynamic parameters: maximal detrusor pressure (MDP) control ≤40 cmH2O.

Success Rate (%)	Achievement of Baseline MDP ≤ 40 cmH_2_O	Achievement of Follow-up 3 MDP ≤ 40 cmH_2_O
Low-dose Oxybutynin	44.6	54.8
High-dose Oxybutynin	36.7	66.7
Oxybutynin + Trospium	16.7	66.7

## Data Availability

The data set used and analyzed during the current study is available from the corresponding author upon reasonable request.
